# Sequencing, Assembly, and Comparative Evolutionary Analysis of the Chloroplast Genome of Kenaf (*Hibiscus cannabinus* L.)

**DOI:** 10.3390/genes16121519

**Published:** 2025-12-18

**Authors:** Ziyi Zhu, Juan Liu, Shenyue Tang, Qingqing Ji, Xingcai An, Junyuan Dong, Xiahong Luo, Changli Chen, Tingting Liu, Lina Zou, Shaocui Li, Mingbao Luan, Xia An

**Affiliations:** 1 College of Carbon Neutrality, College of Environment and Resources, Zhejiang A&F University, Hangzhou 311300, China; 13456319193@163.com (Z.Z.); liujuan@zafu.edu.cn (J.L.); 13587929160@163.com (S.T.); 2 Zhejiang Xiaoshan Institute of Cotton & Bast Fiber Crops, Zhejiang Institute of Landscape Plants and Flowers, Zhejiang Academy of Agricultural Sciences, Hangzhou 311251, China; jiqingqing1001@163.com (Q.J.); xcan2001str@163.com (X.A.); 13964552682@163.com (J.D.); luoxh@zaas.ac.cn (X.L.); chenchangli@zaas.ac.cn (C.C.); liutt@zaas.ac.cn (T.L.); zoulina1991@yeah.net (L.Z.); lishaocui@zaas.ac.cn (S.L.); 3 School of Agriculture, Yunnan University, Kunming 650500, China; 4 Key Laboratory of Bast Fiber Biology and Processing, Ministry of Agriculture and Rural Affairs, Institute of Bast Fiber Crops, Chinese Academy of Agricultural Sciences, Changsha 410000, China

**Keywords:** kenaf (*H. cannabinus*), chloroplast genome, genome structure, comparative analysis, phylogeny, molecular markers

## Abstract

Background: Kenaf (*Hibiscus cannabinus* L.) is an important fiber crop belonging to the genus *Hibiscus* in the Malvaceae family. Research on its chloroplast genome holds significant importance for deciphering the evolutionary relationships of the *Hibiscus* species, developing genetic markers, and promoting kenaf (*H. cannabinus*) genetic breeding. Methods: Based on high-throughput sequencing technology, this study completed the sequencing and assembly of the kenaf (*H. cannabinus*) chloroplast genome. Results: (1) The kenaf (*H. cannabinus*) chloroplast genome exhibits a typical circular quadripartite structure with a total length of 163,019 bp, including a large single-copy region (LSC) of 90,467 bp, a small single-copy region (SSC) of 19,486 bp, and a pair of inverted repeat regions (IRa/IRb) of 26,533 bp each. The total GC content is 36.62%, among which, the IR region has the highest GC content (42.61%) and the SSC region the lowest (30.87%). (2) A total of 131 genes were annotated, including 85 mRNAs, 37 tRNAs, 8 rRNAs, and 1 pseudogene. Their functions cover photosynthesis (e.g., *pet* and *atp* family genes), self-replication (e.g., *rpl*, *rps*, and *rpo* family genes), and genes with unknown functions (e.g., *ycf1* and *ycf2*). A codon usage bias analysis revealed that the relative synonymous codon usage (RSCU) value of the stop codon UAA is the highest (1.6329), and codons ending with A/U are preferentially used (e.g., GCU for alanine with RSCU = 1.778). (3) A repeat sequence analysis identified various interspersed repeat sequences (predominantly 30~31 bp in length, with a relatively high proportion in the 30~40 bp range, including forward and palindromic types) and simple sequence repeats (cpSSRs). Among them, single-base repeat SSRs account for the highest proportion (e.g., (A)8 and (T)9), and specific SSR primers were designed. (4) A comparative evolutionary analysis indicated that the Ka/Ks ratios (nonsynonymous substitution rate/synonymous substitution rate) of core chloroplast genes (e.g., *rps2* and *rpoC2*) in kenaf (*H. cannabinus*) are all less than 1 (0.145~0.415), suggesting that they are under purifying selection. The collinearity similarity of chloroplast genomes between kenaf (*H. cannabinus*) and its closely related species reaches over 99.97%, and the IR region boundaries are relatively conserved. The phylogenetic tree shows that kenaf (*H. cannabinus*) clusters with closely related *Hibiscus* species with a 100% bootstrap value, indicating a close genetic relationship. Conclusions: This study provides basic data for the functional analysis of the kenaf (*H. cannabinus*) chloroplast genome, the phylogeny of *Hibiscus*, and the utilization of genetic resources.

## 1. Introduction

Kenaf (*H. cannabinus*) (2n = 2x = 36), an annual bast fiber crop in the genus *Hibiscus* of the Malvaceae family, is a globally important source of cellulose [1], possessing significant industrial value and ecological adaptability. Its fibers are widely used in traditional textile and papermaking, and have great potential in new building materials [2,3], environmental adsorbents [4], and recycled plastic composites [5,6,7]—making it a multi-functional crop in balancing economic benefits and green development [8]. Moreover, kenaf (*H. cannabinus*) is highly tolerant to abiotic stresses like drought and salinity, and plays an important role in marginal land utilization and ecological restoration [9]. An in-depth analysis of its genetic background is crucial for promoting variety improvement and industrial upgrading [10,11].

Chloroplasts are key and dynamic organelles in plant cells that convert solar energy into carbohydrates through photosynthesis and oxygen release [12,13]. Due to their conserved structure and moderate evolutionary rate, chloroplasts have become ideal models for studying plant phylogeny, species differentiation, and genetic variation [14,15]. For kenaf (*H. cannabinus*), research on the chloroplast genome is also closely related to the mechanism of cytoplasmic male sterility (CMS)—a key basis for the utilization of heterosis in kenaf (*H. cannabinus*). Traditional views hold that CMS is closely associated with the mitochondrial genome, but recent studies have shown that chloroplasts may be involved in CMS formation by regulating photosynthetic efficiency and energy metabolism balance, providing a new perspective for deciphering the fertility regulation mechanism of kenaf (*H. cannabinus*) [16,17,18]. Therefore, clarifying the complete structure and evolutionary characteristics of the kenaf (*H. cannabinus*) chloroplast genome is an important prerequisite for understanding its genetic laws and agronomic trait regulation.

In recent years, progress has been made in research on the chloroplast genomes of kenaf (*H. cannabinus*) and its related species: Tang et al. [19] completed the chloroplast genome sequencing of kenaf (*H. cannabinus*) CMS line P3A and its maintainer line P3B. Among them, nonsynonymous mutations in photosynthesis-related genes (e.g., *atpB*, *rpl20*, and *ycf1*) provided clues for CMS mechanism research; Cheng et al. [20] determined the chloroplast genome of kenaf (*H. cannabinus*) cultivar ‘Fuhong 952′ and found that SSC region orientation variation is widespread among Malvaceae species, with *ycf1* as a highly variable region and *rrn32* as a conserved gene, providing a basis for Malvaceae systematics; Koo et al. [21] corrected the assembly bias of the SSC region in the chloroplast genome of *Hibiscus syriacus* through long-read sequencing, revealing the adaptive evolutionary characteristics of IR contraction/expansion and RNA editing sites in the tribe *Hibisceae*.

However, existing research still has limitations: first, it focuses on a few kenaf (*H. cannabinus*) genotypes, and the exploration of chloroplast genome variation patterns among kenaf (*H. cannabinus*) varieties from different geographical origins and with different uses is insufficient; second, in-depth comparative analysis with more closely related species within the tribe *Hibisceae* (e.g., *Abelmoschus esculentus*, *Talipariti hamabo*) is lacking, and the evolutionary status of kenaf (*H. cannabinus*) needs further clarification; and third, the association between the chloroplast genome variation and important agronomic traits of kenaf (*H. cannabinus*) such as fiber quality and stress resistance has not been thoroughly explored. Although this study only focuses on one kenaf (*H. cannabinus*) genotype and does not fully delve into the association with agronomic traits, improving the completeness of genome assembly and the reliability of variation sites provides higher-quality basic data for subsequent kenaf (*H. cannabinus*) genetic diversity analysis; the preliminary exploration of the molecular mechanism of highly variable genes also builds a bridge for subsequent analysis of the association between chloroplast gene functions and important kenaf (*H. cannabinus*) traits, with significant phased supplementary value. By systematically conducting structural feature comparison, variation hotspot mining, and multi-species phylogenetic analysis, this study contributes to enriching the chloroplast genome resource pool of Malvaceae plants, offers insights to clarify the evolutionary position of kenaf (*H. cannabinus*), and supplies important basic data to support efforts in kenaf (*H. cannabinus*) germplasm genetic diversity conservation, molecular marker development, and the deciphering of CMS fertility regulation mechanisms.

## 2. Materials and Methods

### 2.1. Experimental Materials

The tested kenaf (*H. cannabinus*) cultivar ‘H368′ was grown at the experimental base of the Zhejiang Xiaoshan Cotton and Hemp Research Institute, with the geographical coordinates of 30°4′13″ N and 120°13′34″ E, and an altitude of 7.95 m. The region has an average annual temperature of 16.1 °C, an average annual surface temperature of 18.3 °C, an average annual precipitation of 1402.5 mm, and an average annual sunshine duration of 2006.9 h. The soil is coastal sandy loam formed by reclamation along the Qiantang River in 1993, with a pH value of 7.46.

Select 6 healthy plant leaves, wash to remove impurities, dry to ensure no residue, mix and weigh 2 g of the sample, and seal and store the sample in a pre-sterilized and pre-cooled EP tube. The samples were quickly frozen in liquid nitrogen for 10 min and then stored in a −80 °C refrigerator for later use.

The raw sequencing data of kenaf ‘H368′ (including paired-end reads) and its annotated chloroplast genome (FASTA format + GFF3 annotation) have been submitted to the NCBI GenBank database. The NCBI GenBank is PX646974.

### 2.2. DNA Extraction and Sequencing

Genomic DNA was extracted using the Jisi Huiyuan D312 universal plant DNA extraction kit. The qualified threshold for DNA quality determined in this experiment is as follows: concentration 78.02 ng/μL, volume 40 μL, total amount 3.12 μg, OD260/280 ratio 1.76, OD260/230 ratio 0.89. After the quality of the sample genomic DNA was qualified, the DNA was randomly fragmented using a Covaris ultrasonic disruptor. Subsequently, purification of fragmented DNA, end repair, 3′ end A-tailing, and sequencing adapter ligation were completed in sequence. After screening the target fragment size by agarose gel electrophoresis, PCR amplification was performed to construct a sequencing library. After the library quality inspection was qualified, paired-end (PE) sequencing was carried out on the Illumina NovaSeq X Plus platform, with a sequencing read length of 150 bp.

Raw sequencing data were filtered using the fastp v0.23.4 software (https://github.com/opengene/fastp, accessed on 8 October 2025) [22]: 1. Remove sequencing adapters and primer sequences from the reads. 2. Select reads with an average quality value ≥ Q5. 3. Eliminate reads with empty base content exceeding 5%. High-quality reads (clean data) were obtained after the above quality control.

### 2.3. Chloroplast Genome Assembly and Functional Annotation

To simplify the sequence assembly difficulty, the bowtie2 v2.2.4 (http://bowtie-bio.sourceforge.net/bowtie2/index.shtml, accessed on 10 October 2025) software in very-sensitive-local mode was used to align the self-built chloroplast genome database to extract cpDNA sequences. GetOrganelle v1.7.7.1 (https://github.com/Kinggerm/GetOrganelle, accessed on 10 October 2025) was used for chloroplast genome assembly, with kmer values set to 21, 45, 65, 85, 105, and 125, respectively. The complete command line is as follows: get_organelle_from_reads.py -1 *Hibiscus_cannabinus* L._R1_001.fastq.gz -2 *Hibiscus_cannabinus* L._R2_001.fastq.gz -o *Hibiscus_cannabinus*_L. -t 20 -k 21,45,65,85,105,125 -F embplant_pt -R 10. The assembly process did not rely on a reference genome, and the workflow included seed sequence acquisition, iterative extension, SSPACE contig connection, Gapfiller gap filling, genome correction, and coordinate rearrangement, ultimately obtaining a complete circular genome.

**Figure A1:**
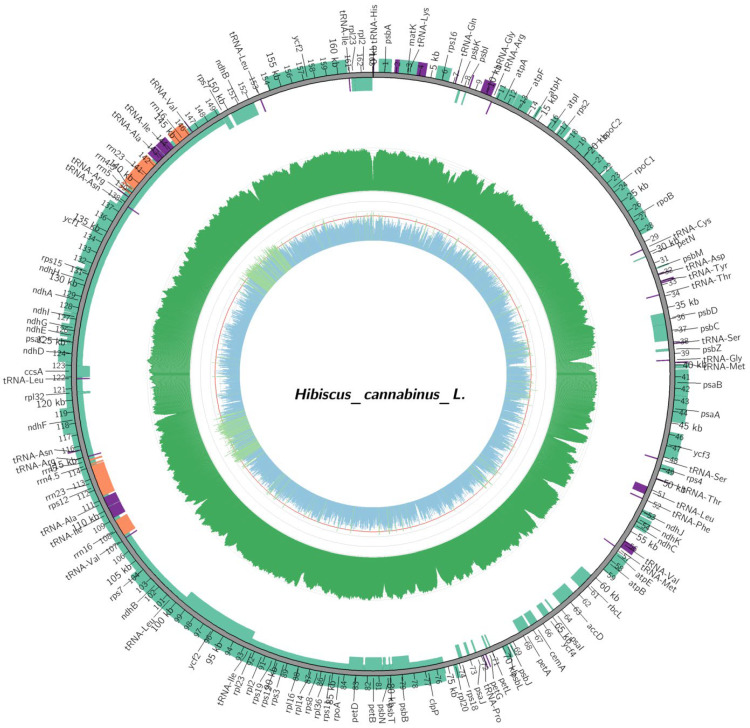
Assembly sequence genome coverage map. Note: 1. The outermost circle represents the genome sequence, with coding genes indicated by green boxes, tRNA by purple boxes, and rRNA by orange boxes. 2. The inner green ring represents coverage depth. 3. The innermost circle represents the GC content of the genome, with segments having a GC content greater than 50% shown in green lines and those with lower content in blue lines.

**Figure A2:**
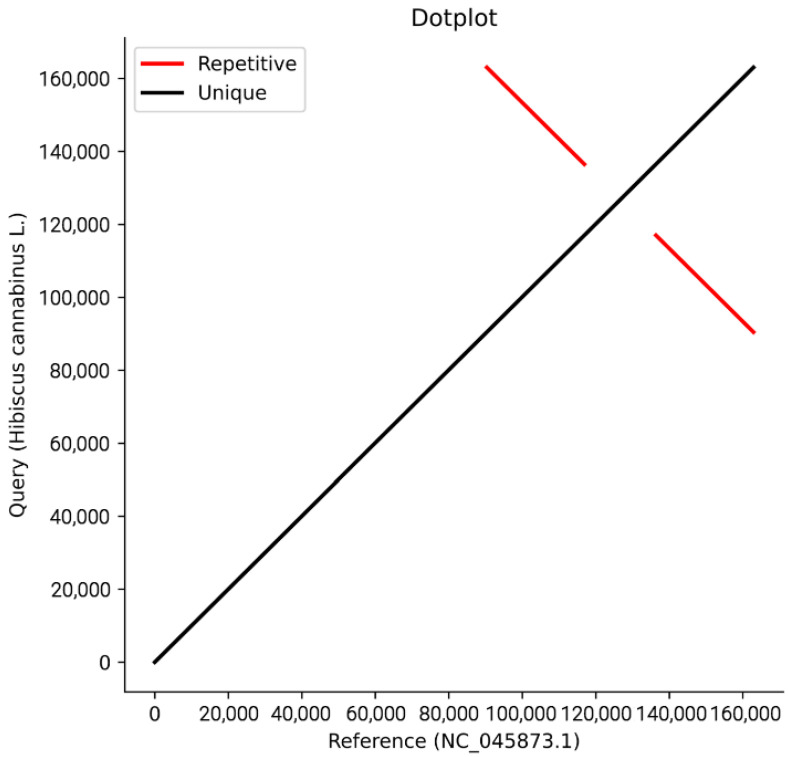
Collinear dotplot plot with reference sequence.

**Figure A3:**
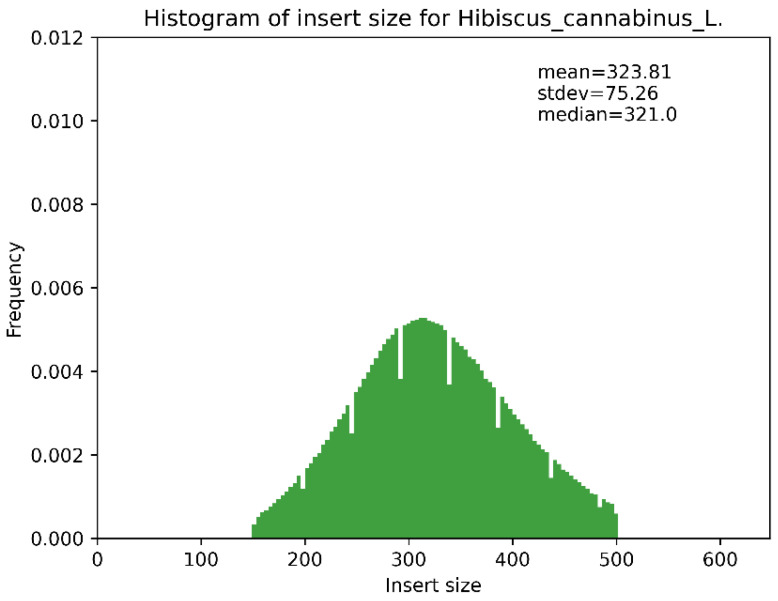
Insert fragment distribution map.

**Figure A4:**
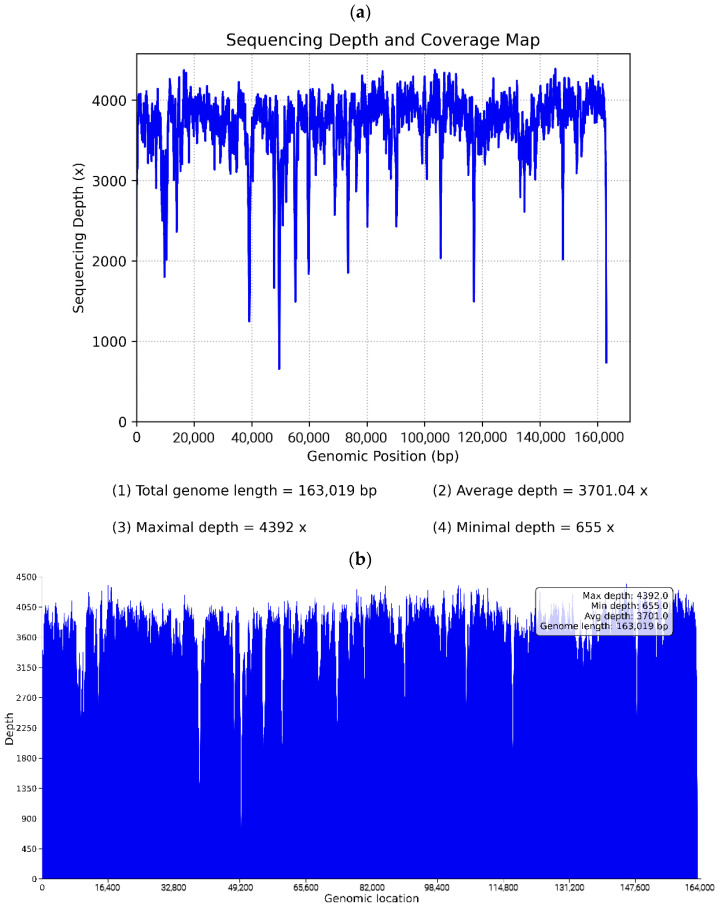
Distribution map of sequencing depth and coverage of chloroplast genome. (**a**) Depth distribution and parameters of genome sequencing, (**b**) Depth map of *H. cannabinus* chloroplast genome sequencing.

The assembly was automatically circularized, generating two complete sequence files (embplant_pt.K125.complete.graph1.1.path_sequence.fasta and embplant_pt.K125.complete.graph1.2.path_sequence.fasta). Ambiguous regions (e.g., homopolymers and IR boundaries) were resolved by the high-quality assembly: the original assembly graph showed a typical quadripartite structure without redundant branches or bubbles, and no long repeat sequences (maximum repeat length was 66 bp, easily spanned by Illumina PE150 reads). All bases were fully covered with overlapping reads, ensuring base accuracy. The appendix has been supplemented with the assembly sequence genome coverage map (Figure A1), collinear dotplot plot with reference sequence (Figure A2), and the insert fragment distribution map (Figure A3), distribution map of sequencing depth and coverage of chloroplast genome(Figure A4) and reads re-sequencing genomic results statistics table (Table A1).

Two methods were used to improve annotation accuracy. 1. The Prodigal v2.6.3 software (https://www.github.com/hyattpd/Prodigal, accessed on 14 October 2025) [23] was used to annotate chloroplast CDS, hmmerv3.1b2 (http://www.hmmer.org/, accessed on 15 October 2025) was used to predict rRNA [24], and aragorn v1.2.38 (http://www.ansikte.se/ARAGORN/, accessed on 15 October 2025) was used to predict tRNA [25]. 2. Published gene sequences of closely related species were downloaded from the NCBI database (https://www.ncbi.nlm.nih.gov/, accessed on 8 October 2025), and the assembled sequences were aligned using blast v2.6 (https://blast.ncbi.nlm.nih.gov/Blast.cgi, accessed on 15 October 2025) [26] to obtain the second annotation result. Use reference sequence NC_045873.1. Manual verification was performed on the differential annotations of the two methods to eliminate errors and redundant annotations, clarify the boundaries of multi-exon genes, and finally determine the annotation results. Start/stop codons and intron–exon boundaries were determined by visual alignment and the verification of annotated genomes and reference sequences in the Geneious software v2023.1. The chloroplast genome map was drawn using the OGDRAW software [27], and gene functions were mainly divided into photosynthesis-related genes, self-replication-related genes, and other functional genes, including some genes with unknown functions.

### 2.4. RSCU Analysis

Uniq CDS was screened (only one copy was retained for multi-copy CDS), and the RSCU value was calculated according to the following formula: (Number of one codon encoding a certain amino acid/Total number of all codons encoding the amino acid)/(1/Number of codon types encoding the amino acid), i.e., the actual usage frequency of the codon/the theoretical usage frequency of the codon.

### 2.5. Interspersed Repeat Sequence Analysis

The Vmatch v2.3.0 software (http://www.vmatch.de/, accessed on 17 October 2025) [28] combined with Perl scripts was used for repeat sequence identification. The parameters were set to a minimum length of 30 bp and a Hamming distance of 3. The identified types included forward, palindromic, reverse, and complementary types, and the quantity and type distribution characteristics of repeat sequences of different lengths were counted.

### 2.6. cpSSR Analysis

The MISA v1.0 software (MIcro SAtellite identification tool, https://webblast.ipk-gatersleben.de/misa/, accessed on 17 October 2025) was used for cpSSR analysis. The parameters were set as single-base repeats ≥ 8 times (1-8), 2-5, 3-3, 4-3, 5-3, and 6-3. SSR markers in the chloroplast genome were analyzed, information such as the type, sequence, and position of SSRs was recorded, and corresponding primers were designed.

### 2.7. Ka/Ks Analysis

Gene sequence alignment was performed using the mafft v7.427 software (https://mafft.cbrc.jp/alignment/software, accessed on 17 October 2025) [29], and the ratio of nonsynonymous substitution rate (Ka)-to-synonymous substitution rate (Ks) was calculated using the KaKs_Calculator v2.0 software (https://sourceforge.net/projects/kakscalculator2/, accessed on 17 October 2025) [30] to analyze the selection pressure on genes (ratio > 1 indicates positive selection and <1 indicates purifying selection).

### 2.8. Nucleotide Diversity and Boundary Analysis

The MAFFT v7.505 software (--auto mode) was used for the full-sequence alignment of homologous gene sequences from different species, and the dnasp5 software [31] was used to calculate the pi value of each gene. A line chart of gene pi values was drawn to intuitively present the nucleotide diversity characteristics.

For boundary analysis, the cloud platform tool CPJSdraw from Jisi Huiyuan (http://cloud.genepioneer.com:9929/#/tool/alltool/detail/296, accessed on 20 October 2025) was used to visually display the information of the 4 boundaries (LSC-IRb, IRb-SSC, SSC-IRa, and IRa-LSC) between IR and LSC and SSC in the chloroplast genome.

### 2.9. Phylogenetic Analysis

For phylogenetic analysis, the chloroplast genomes of 25 Malvaceae plants and 3 outgroup plants (Arabidopsis thaliana, Glycine max, and Morus alba) were selected as references, and all data were downloaded from the NCBI database (https://www.ncbi.nlm.nih.gov/, accessed on 20 October 2025). The PhyloSuite v1.2.3 software was used to screen the shared genes among all sequences. After multiple sequence alignment using the MAFFT v7.427 software (--auto mode) [32], the trimAl (v1.4.rev15) software [33] was used to remove regions with low alignment reliability, and the species CDS sequences were concatenated. Under the Bayesian information criterion, the jModelTest v2.1.10 software was used to select the optimal nucleotide substitution model. The RAxML v8.2.10 software (https://cme.h-its.org/exelixis/software.html, accessed on 25 October 2025) [34] was used to construct a maximum likelihood phylogenetic tree with the GTRGAMMA model and 1000 rapid bootstrap analyses.

### 2.10. Sequence Homology Collinearity Analysis

The Mauve software v 2.3.1 (https://darlinglab.org/mauve/mauve.html, accessed on 25 October 2025) [35] was used to carry out genome alignment with default parameters to visually present local collinear blocks (LCBs) and collinearity relationships. Among them, colored blocks represent homologous regions, the position of blocks relative to the center line (upper and lower) reflects the sequence alignment direction, and the similarity map within the blocks reflects the sequence similarity level.

## 3. Results

### 3.1. Gene Structure Annotation

Table 1 shows the statistical information of the kenaf (*H. cannabinus*) chloroplast genome annotation, including 131 genes, 85 mRNAs, 37 tRNAs, 8 rRNAs, and 1 pseudogene (coordinates: 116029-117039). The pseudogene may be generated due to incomplete replication of the normal copy of ycf1 in the junction region of IRa and SSC, and similar mutations have been found in the CP genomes of other angiosperm species [36,37].

Table 2 presents the functional classification of kenaf (*H. cannabinus*) chloroplast genome genes, dividing the genes into four core functional modules: first, photosynthesis-related genes (44), including photosystem II subunits (e.g., *psbA* and *psbB*), cytochrome b/f complex subunits (*petA* and *petB*, etc.), ATP synthase subunits (*atpA* and *atpB*, etc.), as well as photosystem I subunits, NADH dehydrogenase subunits, and rubisco large subunits. Among them, genes such as *ndhB* have two copies, which are key gene clusters for kenaf (*H. cannabinus*) to achieve light energy conversion. Second, self-replication-related genes (74), including ribosomal large/small subunit proteins (*rpl14* and *rps11*, etc.), RNA polymerase subunits (*rpoA* and *rpoB*, etc.), as well as all 8 rRNAs (including multiple copies) and 37 tRNAs. Some ribosomal proteins have two copies, supporting the replication and genetic information transmission of the chloroplast genome. Third, other functional genes (6), such as maturase *matK*, protease *clpP*, and translation initiation factor *infA*, etc., involved in gene maturation, protein metabolism regulation, and translation initiation processes. Fourth, genes with unknown functions (6), mainly conserved hypothetical open reading frames such as *ycf1* and *ycf2*, reserving research space for subsequent functional mining.

### 3.2. Chloroplast Genome Map

**Figure 1:**
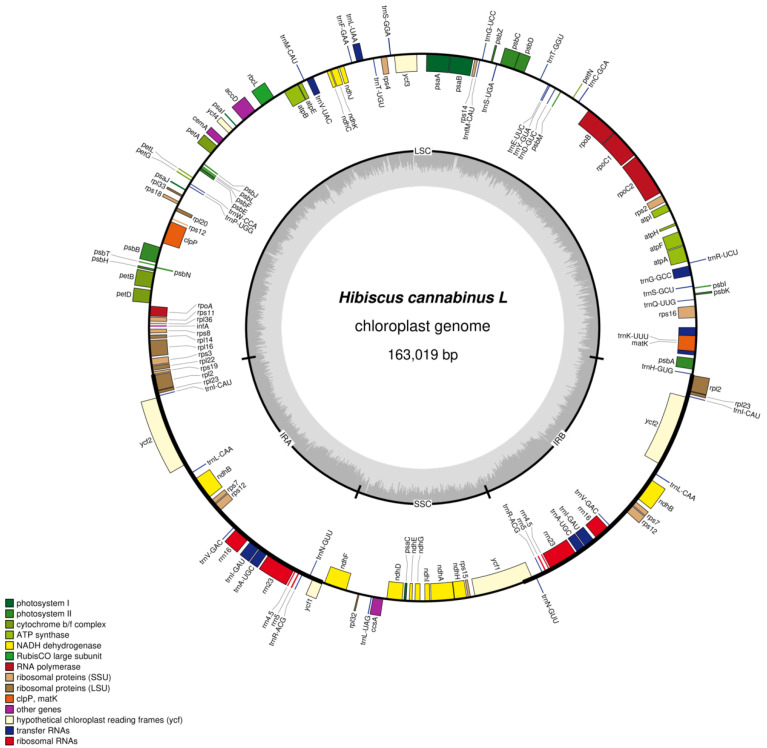
Chloroplast genome map. Note: genes encoded on the forward strand are displayed on the outer circle, while those encoded on the reverse strand are shown on the inner circle. The gray inner ring represents GC content distribution.

The chloroplasts of kenaf (*H. cannabinus*) show typical tetragonal characteristics in the circular structure (Figure 1, Table 3): the outer circular track corresponds to the full-length 163,019 bp sequence of the genome, and the inner part clearly distinguishes the large single-copy region (LSC, 90,467 bp), small single-copy region (SSC, 19,486 bp), and a pair of inverted repeat regions (IRa/IRb, 26,533 bp each) through regional division. Among them, IRa and IRb are symmetrically distributed on the upper and lower sides of the map, and their inverted repeat characteristics are reflected by the consistent annotation of genes (such as rRNA genes *rrn16* and *rrn23*) in the region, which is consistent with the highly conserved feature of the IR region with a GC content of 42.61%.

In terms of gene annotation, Figure 1 clarifies the gene distribution and coding direction through “inner and outer tracks + arrow direction”; forward coding genes (such as photosynthesis-related *psbA*, *rbcL*, and *atpA*) are located on the outer track, and reverse coding genes (such as *ndhF* and *ycf1*) are located on the inner track. Functional related genes are clustered—photosynthesis genes are concentrated in the LSC region to form a “photosynthetic gene cluster”, and self-replication-related rRNA genes only exist in the IR region and are symmetrically arranged in double copies, which fully corresponds to the functional classification results of the 131 genes (85 mRNAs, 37 tRNAs, 8 rRNAs, and 1 pseudogene) in the following table.

In addition, the innermost gray track of the map reflects the GC content difference through the color depth gradient. The IR region shows a continuous dark band due to the enrichment of rRNA genes (GC = 42.61%), while the LSC/SSC regions show light bands due to the enrichment of A/T (LSC GC = 34.35%, SSC GC = 30.87%, Table 4).

### 3.3. RSCU Analysis

**Figure 2:**
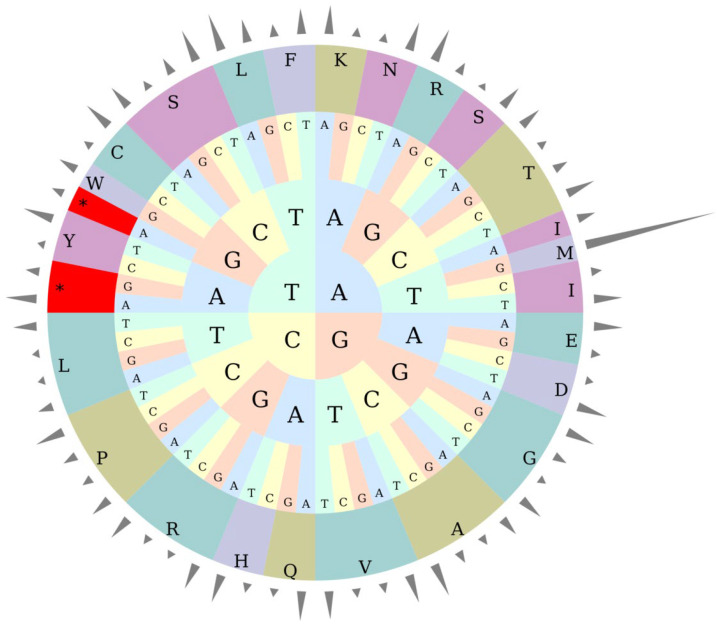
RSCU pie chart. Note: The height of the outermost cylinder is RSCU value, the inner layer is amino acid, and the innermost three layers are codons. * represents the termination codon.

Table 5 and Figure 2 systematically present the codon usage quantity and relative synonymous codon usage (RSCU) of 20 amino acids and stop codons (Ter) in the kenaf (*H. cannabinus*) chloroplast genome. From the core characteristics, among the stop codons, UAA has the highest RSCU value (1.6329), and its usage frequency is significantly higher than that of UAG (0.7215) and UGA (0.6456), making it the preferred translation termination signal for kenaf (*H. cannabinus*) chloroplast genes; the codon preference of amino acids varies significantly. For example, codons such as GCU (1.778) for alanine (Ala), UUA (1.9944) for leucine (Leu), and UGU (1.5354) for cysteine (Cys) have RSCU > 1, which are highly preferred codons, while GCG (0.5208) for Ala and UGC (0.4646) for Cys have RSCU < 1, with low usage frequency; methionine (Met) shows an extreme codon selection characteristic, with only AUG actually used (quantity 528, RSCU = 7), and other codons (AUA, AUC, etc.) have a usage quantity of 0.

In addition, 26 out of 28 highly preferred codons (RSCU > 1) end with A/U, which is consistent with the base composition characteristic of A/T enrichment in the kenaf (*H. cannabinus*) chloroplast genome. It is speculated that this preference can accelerate the synthesis of photosynthesis-related proteins by improving the pairing efficiency of tRNA anticodons, adapting to the high photosynthetic demand of kenaf (*H. cannabinus*) as a fiber crop.

### 3.4. Interspersed Repeat Sequences

Interspersed repeat sequences are another type of repeat sequence different from tandem repeat sequences, distributed in a scattered manner in the genome [38].

**Figure 3:**
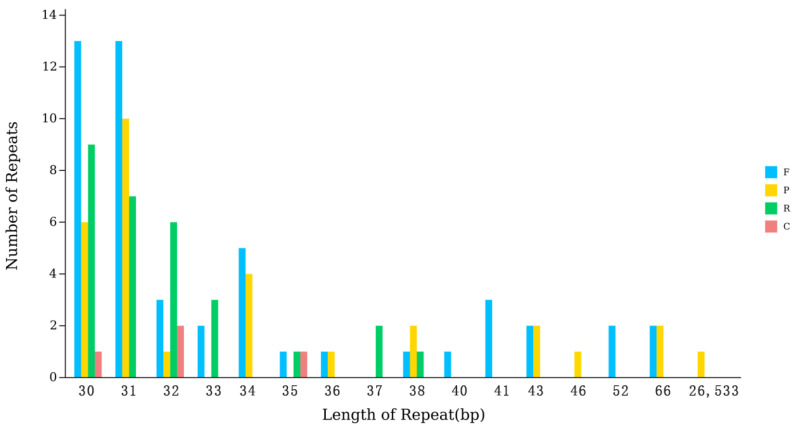
Scatter plot of interspersed repeat sequence Statistics.

As shown in Figure 3, the repeat sequences of the kenaf (*H. cannabinus*) chloroplast genome are highly concentrated in the short-fragment range of 30~31 bp. Among them, there are 29 repeat sequences of 30 bp in length (including F = 13, P = 6, R = 9, C = 1) and 30 repeat sequences of 31 bp in length (including F = 13, P = 10, R = 7, C = 0), accounting for 52.68% of the total repeat sequences (112); the number of repeat sequences in the 32~66 bp length range shows a significant decreasing trend with the increase in length (12 for 32 bp, 5 for 33 bp, 9 for 34 bp, and 4 for 66 bp), and only 1 repeat sequence has a length of 26,533 bp. Combined with the quadripartite structure of the kenaf (*H. cannabinus*) chloroplast genome (Ira/Irb each 26,533 bp), it is speculated that this long-fragment repeat is the inverted repeat core unit of the IR region. In terms of type distribution, forward repeats (F) are the most numerous (49), accounting for 43.75% of the total repeats, followed by reverse repeats (R, 29, 25.89%), palindromic repeats (P, 30, 26.79%), and complementary repeats are the least (4, 3.57%); various repeats are distributed in short-fragment repeats (30~31 bp), while long-fragment repeats (26,533 bp) are only palindromic repeats, reflecting the length correlation of the type distribution.

The core characteristics of the kenaf (*H. cannabinus*) chloroplast interspersed repeat sequences are short-fragment enrichment and long-fragment specificity. Short-fragment repeats (30~31 bp) may be involved in the recombination regulation and transcriptional regulation of intergenic regions, while long-fragment repeats (26,533 bp) provide support for maintaining the structural stability of the IR region of the chloroplast genome. Together, they constitute the functional differentiation pattern of kenaf (*H. cannabinus*) chloroplast repeat sequences [39].

### 3.5. cpSSR

**Figure 4:**
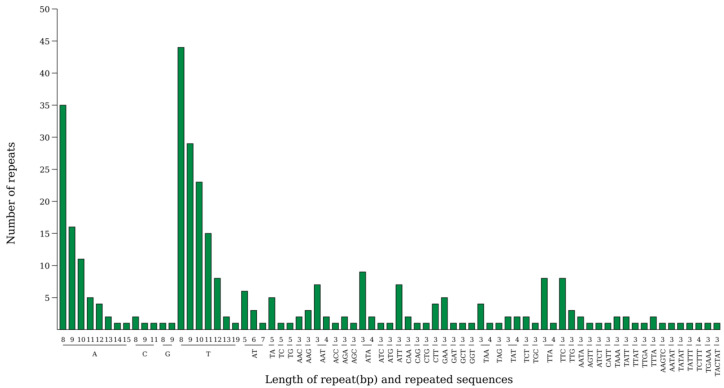
Statistical chart of the number of each type of SSR. Note: The horizontal axis represents SSR repeating units, and the vertical axis represents the number of repeating units.

Figure 4 is a distribution diagram of simple sequence repeat (cpSSR) types in the kenaf (*H. cannabinus*) chloroplast. Among them, single-base repeats (p1) are dominant, with 203 p1-type cpSSRs, accounting for 62.1% of the total cpSSR loci (327); trinucleotide repeats (p3) are the second-most common, with 87 p3-type cpSSRs, accounting for 26.6%. In Figure 4, the column height of the p3-type is second only to that of p1; the number of dinucleotide (p2, 17), tetranucleotide (p4, 13), pentanucleotide (p5, 6), and hexanucleotide (p6, 1) repeat types is very small, accounting for only 11.3% in total, showing a cpSSR type pattern of “single-base dominated, trinucleotide supplemented”.

In terms of the base composition of repeat units, p1-type cpSSRs are dominated by A/T repeats, with only a few G/C repeats, which is consistent with the base composition characteristic of A/T enrichment in the kenaf (*H. cannabinus*) chloroplast genome (the total A + T content of the whole genome is 63.38%). The repeat units of p3-type cpSSRs mostly contain A/T, further confirming the base composition preference of chloroplast cpSSRs.

### 3.6. Ka/Ks Analysis

Table 6 calculates the ratio of nonsynonymous substitution rate (Ka)-to-synonymous substitution rate (Ks) of four core protein-coding genes (*rps2*, *rpoC2*, *rpoC1*, and *rpoB*) in kenaf (*H. cannabinus*) using the MLWL method with reference to the chloroplast genome of a closely related species with NCBI accession number MK792856.1, quantifying the intensity of the selection pressure on genes. The results show that the Ka/Ks values of the four genes are all less than 1, ranging from 0.145 to 0.415, and the *p* values are all less than 0.01, with extremely significant differences. This indicates that these genes are under strong purifying selection during evolution, and natural selection preferentially eliminates harmful nonsynonymous mutations to maintain the stability of core gene functions.

From the perspective of gene function, *rps2* has the lowest Ka/Ks value (0.145) and the greatest purifying selection pressure. Its encoded protein is involved in ribosome assembly and is a basic unit of protein synthesis. Mutations are likely to lead to abnormal translation functions, so it is highly conserved during evolution; *rpoC2* has the highest Ka/Ks value (0.415), but it is still much less than 1. It is speculated that although it may accumulate a small number of adaptive mutations, the core transcriptional function has not differentiated, which is consistent with the conservative characteristics of the chloroplast transcriptional system. This table provides direct quantitative evidence for the evolutionary conservation of kenaf (*H. cannabinus*) chloroplast genes and lays a data foundation for subsequent comparison of selection pressure among *Hibiscus* species.

**Figure 5:**
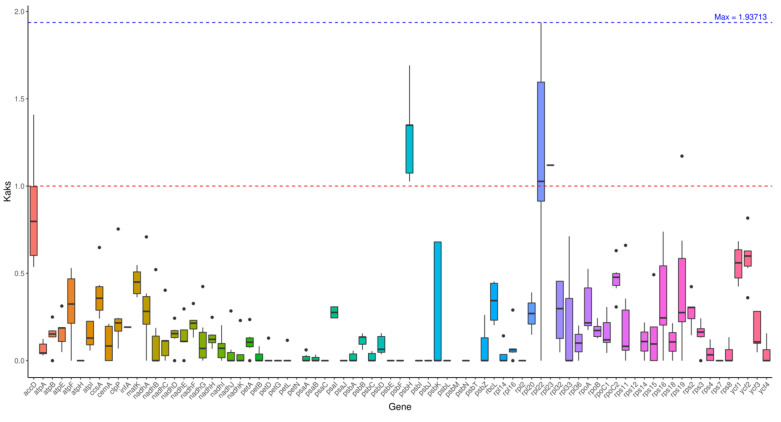
Boxplot of Ka/Ks among species. Note: The x-axis represents gene names, and the y-axis represents kaks values. In the box plot, the upper and lower endpoints of the vertical lines above and below the rectangle indicate the upper and lower bounds of the data, respectively. The thick line inside the rectangle represents the median. The upper and lower edges of the rectangle represent the upper and lower quartiles. The dots outside the upper and lower bounds of the data represent outliers.

Figure 5 intuitively presents the distribution characteristics of the Ka/Ks values of the four genes in Table 6 in the form of a boxplot. The upper and lower edges of the box represent the upper and lower quartiles (0.18~0.40), the thick line inside the box is the median (0.26), and the upper and lower whiskers correspond to the upper and lower edges of the data (0.145~0.415), with no outlier distribution.

The Ka/Ks values of the four genes are all concentrated in the 0.1~0.5 interval, with no data deviation, indicating that the intensity of selection pressure on the core genes of the kenaf (*H. cannabinus*) chloroplast tends to be consistent, and there is no positive selection signal related to functional differentiation; at the same time, the height of the box is relatively narrow (interquartile range 0.22), reflecting that the difference in selection pressure among genes is small, further confirming the overall evolutionary conservation of the coding region of the kenaf (*H. cannabinus*) chloroplast.

### 3.7. Nucleotide Diversity (pi) Analysis

**Figure 6:**
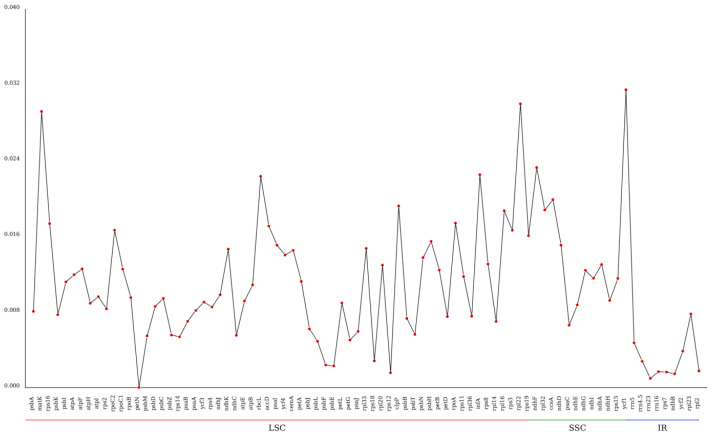
Gene pi value line chart. Note: The horizontal axis represents the gene name, and the vertical axis represents the pi value.

Figure 6 reveals that the nucleotide diversity of the kenaf (*H. cannabinus*) chloroplast genome exhibits distinct regional differentiation, following the pattern of ‘SSC region > LSC region > IR region’—consistent with higher plant chloroplast genome characteristics, where the IR region accumulation has less variation due to inverted repeat gene conversion repair, while the SSC region has more active variation under relatively loose selection pressures [40].

The IR region shows low gene pi values (average 0.001~0.004), with only a few genes having minor variation (pi ≈ 0.003), reflecting its high evolutionary conservation and critical role in maintaining chloroplast genome structural stability.

The pi values of genes in the LSC region (large single-copy region) are generally higher than those in the IR region, with an average of 0.005~0.02, and the variation among genes is obvious: the pi values of most photosynthesis-related genes (such as *psbA* and *atpB*) are low (0.006~0.012), reflecting the conservation of core photosynthetic functions; while the pi values of some intergenic region-associated genes and edge genes (such as tRNA gene *trnH*-GUG and ribosomal protein gene *rps19*) exceed 0.03 (0.049 and 0.031, respectively), becoming high-variation hotspots in the LSC region. It is speculated that these regions are under weak selection pressure and are more likely to accumulate mutations.

The SSC region (small single-copy region) is a relatively active region for variation in the kenaf (*H. cannabinus*) chloroplast genome, with an average gene pi value of 0.018, significantly higher than other regions: among them, the *ycf1* gene has the highest pi value (0.034), followed by the *ndhF* gene (0.027). These two genes are representative genes in the SSC region—although the function of *ycf1* is unknown, it is located near the IR/SSC boundary and is prone to accumulate variation due to boundary expansion and contraction and *ndhF* is involved in the composition of the NADH dehydrogenase complex and may accumulate a small number of adaptive mutations during the adaptive evolution of species. In addition, the pi values of genes such as *ndhD* and *ccsA* in the SSC region are also at a high level (0.015~0.022), further confirming the high-variation characteristics of the SSC region.

Through the line distribution in Figure 6, three highly variable genes (pi ≥ 0.03) can be further screened out, namely *trnH-GUG* (0.049) and *rps19* (0.031) in the LSC region and *ycf1* (0.034) in the SSC region. The high-variation characteristics of these genes make them high-resolution molecular markers for genetic diversity analysis of kenaf (*H. cannabinus*) germplasm resources, variety identification, and differentiation of closely related *Hibiscus* species; while the low-variation genes in the IR region can be used as reliable targets for phylogenetic analysis of higher taxonomic units (such as *Hibiscus*).

### 3.8. Boundary Analysis

Chloroplast genome boundaries (JLB, JSB, JSA, and JLA) between inverted repeats (IRa/IRb), large single-copy (LSC), and small single-copy (SSC) regions are core mechanisms driving genome structural variation and influencing stability, as well as key targets for deciphering species evolutionary traits [41,42].

**Figure 7:**
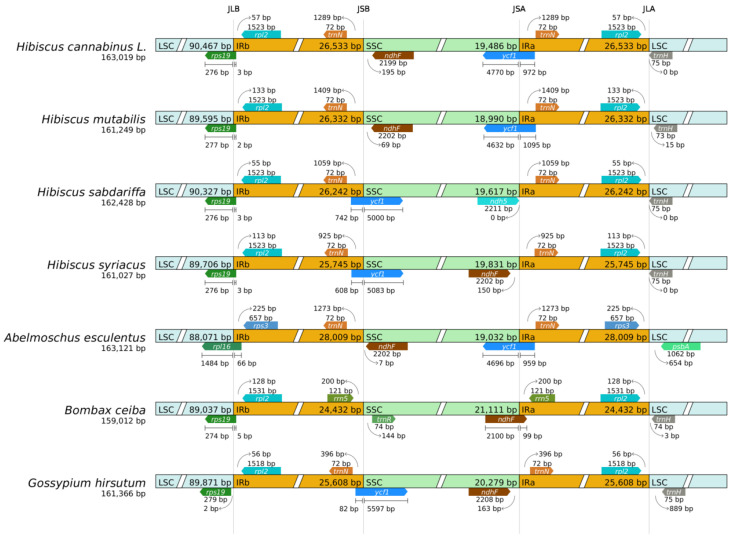
Analysis of chloroplast IR boundary changes. Note: The thin lines represent the connection points of each region, and the information of genes near the connection points is presented on the map.

The IR boundary visualization analysis in Figure 7 shows that the four boundaries of the kenaf (*H. cannabinus*) chloroplast genome present clear and stable structural characteristics: the JLB (LSC-IRb) boundary runs through the ribosomal protein gene *rps19*, with most of its 5′ end fragments (276 bp) located in the LSC region and a short fragment (15 bp) at the 3′ end extending into the IRb region, forming a typical cross-region distribution pattern; the JSB (IRb-SSC) and JSA (SSC-IRa) boundaries are strictly symmetrically distributed, both marked by the conserved hypothetical open reading frame gene *ycf1*. The lengths of the fragments extending into the SSC region on both sides are 51 bp, and the lengths of the fragments located in the IR region are 1110 bp. This symmetry directly reflects the structural stability of the inverted repeat sequences in the IR region; and the tRNA gene trnH-GUG near the JLA (IRa-LSC) boundary is completely located in the LSC region, with a distance of about 75 bp from the boundary, and no cross-region phenomenon, ensuring the stable execution of amino acid transport functions [43]. These boundary characteristics are highly consistent with the typical structure of the chloroplast genome of *Hibiscus* plants, with no gene breakage or pseudogenization, reflecting the evolutionary conservation of genes related to the core physiological functions of kenaf (*H. cannabinus*).

Combined with the boundary comparison of closely related *Hibiscus* species (*Hibiscus mutabilis*, *Hibiscus sabdariffa*, and *Hibiscus syriacus*, etc.) in Figure 7, in terms of conservation, the boundary marker genes of closely related species are consistent with those of kenaf (*H. cannabinus*), all defining the four boundaries with *rps19*, *ycf1*, and trnH-GUG. The difference in the length of the cross-region fragments of *rps19* and *ycf1* is only 1–3 bp, and the difference in the total length of the IR region is only 3–5 bp, much lower than the common variation range of the IR region in higher plants. This is consistent with the highly conserved characteristics of the IR region in the chloroplast genome of *Hibiscus* species such as *Hibiscus* mutabilis [44]. Local microvariations are only reflected in the distance between trnH-GUG and the boundary at the JLA boundary: 75 bp for kenaf (*H. cannabinus*), 72 bp for *Hibiscus syriacus*, and 78 bp for *Hibiscus mutabilis*, with a fluctuation range ≤ 6 bp, no functional impact, and it is speculated to be the accumulation of neutral mutations during species differentiation. This stable boundary structure is the result of purifying selection during the long-term evolution of *Hibiscus* plants. It ensures the stability of core functions such as photosynthesis and ribosome assembly, while local microvariation characteristics provide potential targets for the screening of interspecific molecular markers.

### 3.9. Phylogenetic Analysis

In this study, a phylogenetic tree was constructed based on the shared CDS sequences of the chloroplast genomes of kenaf (*H. cannabinus*), 25 Malvaceae plants (including closely related *Hibiscus species*), and 3 outgroup plants (*Arabidopsis thaliana*, *Glycine max*, and *Morus alba*).

**Figure 8:**
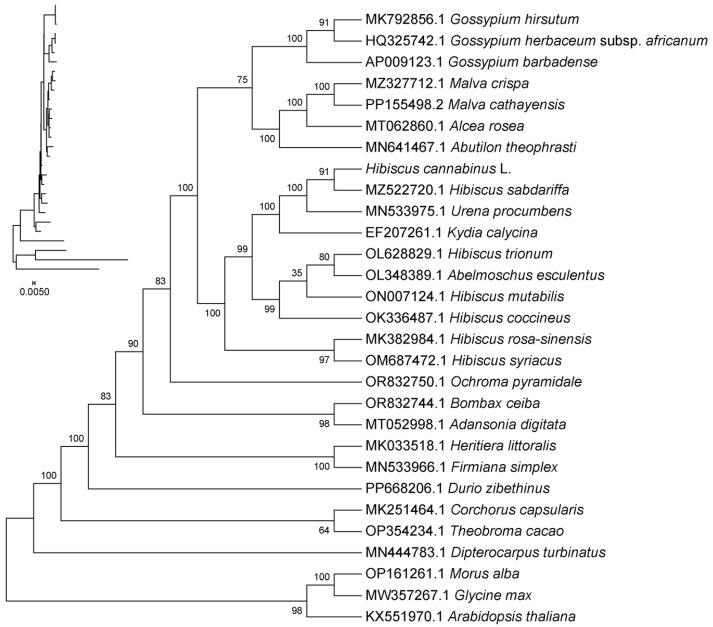
Chloroplast system evolutionary analysis. Note: (1) The name of the sequence is the species’ Latin name. (2) Evolutionary branch length: Also known as genetic variation degree or evolutionary distance. It represents the extent of change in the evolutionary branch; the shorter it is, the smaller the difference and the closer the evolutionary distance. (3) Distance scale: The unit length representing the numerical difference between organisms or sequences, equivalent to the scale of the evolutionary tree. (4) Bootstrap value: Used to show the credibility of the branches in the evolutionary tree. It is usually represented by a number between 0 and 100.

The results in Figure 8 show that kenaf (*H. cannabinus*) clusters with *Hibiscus* species (*Hibiscus sabdariffa*, *Hibiscus mutabilis*, and *Hibiscus syriacus*, etc.) into an independent branch with a 100% bootstrap support rate. The evolutionary distance within the branch is short (the evolutionary distance between kenaf (*H. cannabinus*) and *H. sabdariffa* is only 0.002), indicating a close genetic relationship, and it is speculated that they originated from recent differentiation of the same ancestor; this branch is adjacent to the branches of *Gossypium* (*Gossypium hirsutum* and *Gossypium herbaceum*) and *Abelmoschus* (*Abelmoschus esculentus*) with a bootstrap support rate of 95%, reflecting the close relationship between *Hibiscus* and these two genera within Malvaceae, while the evolutionary distance from Bombax (*Bombax ceiba*) and Durio (*Durio zibethinus*) is relatively far, clearly presenting the differentiation pattern at the generic level within Malvaceae.

The outgroup plants (*Arabidopsis thaliana*, *Glycine max*, and *Morus alba*) in Figure 8 are located at the base of the phylogenetic tree, and the evolutionary distance from Malvaceae plants is significantly greater than that between species within the family (branch length 0.005), verifying the rationality of the phylogenetic tree structure; at the same time, the evolutionary distance between other species in the *Hibiscus* branch (such as *Hibiscus trionum* and *Hibiscus coccineus*) and kenaf (*H. cannabinus*) is slightly greater than that between kenaf (*H. cannabinus*) and *H. sabdariffa*, further clarifying the specific evolutionary position of kenaf (*H. cannabinus*) within *Hibiscus*.

### 3.10. Chloroplast Sequence Homology and Collinearity Analysis

**Figure 9:**
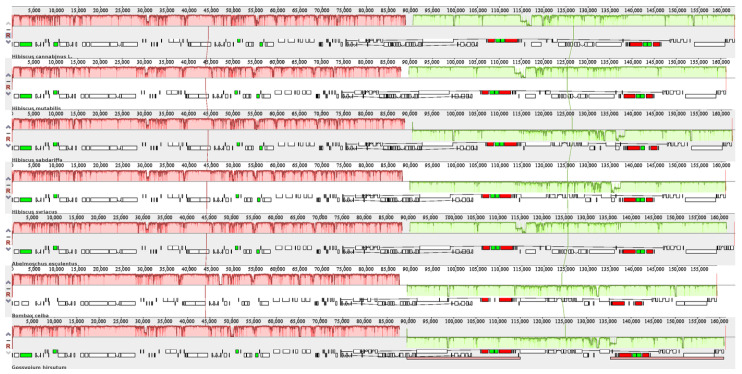
Chloroplast sequence homology analysis. Note: The short blocks represent gene positions in the genome, where white indicates CDS (Coding DNA Sequence), green indicates tRNA, and red indicates rRNA. The colored blocks (Locally Collinear Blocks, LCBs) represent regions that are aligned and matched with a part of another genome, indicating homology. When a block is located above the central line, the aligned region is in the forward orientation relative to the first genome sequence. Blocks below the central line indicate reverse-complementary alignment. Regions outside the blocks represent areas where homology is lacking between the input genomes. Within each block, Mauve plots a similarity profile of the genome sequences. The height of the similarity graph corresponds to the average level of similarity in that region of the genome sequences. Lines connecting the colored blocks represent collinearity relationships.

From Figure 9, it can be seen that the chloroplast genomes of Malvaceae species are generally highly conserved, with coding regions (such as *rbcL* and *atpB*) showing significant red high conservation characteristics, while non-coding regions (gene spacer regions) are light green variation regions. The genome sequence, gene arrangement, and IR region (reverse repeat region) boundary of kenaf and *Hibiscus* species are completely collinear, without large segment rearrangement or structural loss, which confirms that the assembly structure of the kenaf chloroplast genome is accurate and conforms to the conservation and evolution characteristics of the Malvaceae chloroplast genome.

## 4. Discussion

Based on the Illumina NovaSeq X Plus platform, this study sequenced and analyzed the chloroplast genome of kenaf (*H. cannabinus*) and carried out reference-free genome assembly using the GetOrganelle software, finally obtaining a complete circular genome with a length of 163,019 bp and a GC content of 36.62%.

Compared with previous research results, Tang et al. [19] determined that there is a 237 bp length difference between the chloroplast genomes of kenaf (*H. cannabinus*) CMS line P3A (163,360 bp) and maintainer line P3B (163,597 bp), and Cheng et al. [20] reported that the length of the chloroplast genome of kenaf (*H. cannabinus*) ‘Fuhong 952′ is 162,903 bp. The genome length assembled in this study is similar to the above results but has slight differences. This difference may be related to the specificity of kenaf (*H. cannabinus*) genotypes or the different assembly strategies. In addition, this study verified the assembly reliability through multiple quality control methods such as reads mapping, collinearity analysis with the reference sequence NC_045873.1, and gene quantity comparison (consistent number of mRNAs, tRNAs, and rRNAs), further excluding the impact of assembly errors on genome length differences, and providing high-quality basic data for subsequent research on the kenaf (*H. cannabinus*) chloroplast genome.

A total of 85 mRNAs, 37 tRNAs, 8 rRNAs, and 1 pseudogene were identified in this study. Gene function classification shows that photosynthetic system-related genes and self-replication-related genes are all present completely and also include conserved open reading frames with unknown functions such as *ycf1* and *ycf2*. This annotation result is slightly different from the chloroplast gene composition of kenaf (*H. cannabinus*) ‘Fuhong 952′ reported by Cheng et al. [20] (79 mRNAs, 30 tRNAs, and 4 rRNAs). It is speculated that this difference may be related to the parameter setting differences of annotation tools or the gene composition specificity of different kenaf (*H. cannabinus*) genotypes; compared with the phenomenon found by Tang et al. [19] that the CMS line P3A lacks two protein-coding genes *rps19*-D2 and petN and adds three tRNA genes, the core genes of the chloroplast genome of different kenaf (*H. cannabinus*) genotypes in this study have strong conservation, and the gene deletion of the CMS line may be specifically related to the fertility regulation process.

Simple sequence repeats (SSRs) are important molecular markers for plant genetic diversity analysis, molecular breeding, and variety identification [45]. Compared with the kenaf cpSSR (38%) reported by Cheng [20], the high proportion of single-base A/T duplication (62.1%) in this study is actually in coordination with the base preference of kenaf chloroplast A + T enrichment (63.38%). The enrichment of this type of duplication in the transcriptional spacer can enhance the flexibility of gene expression and regulation and provide a genetic basis for kenaf to rapidly adjust the transcription efficiency of photosynthetic genes when responding to different environmental lights. The newly identified cpSSR loci in this study—particularly intergenic single-base A/T tandem repeat loci—contribute to enriching kenaf’s (*H. cannabinus*) molecular marker library and provide potentially applicable tools for evaluating genetic diversity, identifying variety purity, and analyzing genetic relationships among kenaf varieties.

In terms of the comparative evolutionary analysis, the *rps2*, *rpoC2*, *rpoC1*, and *rpoB* are all less than 1, indicating that these genes are under strong purifying selection during evolution. This trend is consistent with the purifying selection characteristics of the *ndhD* gene found by Koo et al. [21] in the tribe *Hibisceae*, indicating that these genes play a key role in maintaining the basic functions of chloroplasts, and their sequence variation may be subject to strict functional constraints; the nucleotide diversity (pi) analysis identified multiple highly variable gene regions, providing candidate regions for the subsequent development of molecular barcodes for Malvaceae species.

The IR boundary analysis revealed the differences in expansion and contraction of the LSC-IRb and SSC-IRa boundaries between kenaf (*H. cannabinus*) and *Hibiscus syriacus*, *Abelmoschus esculentus*, supplementing the details of the dynamic evolution of the IR region in the chloroplast genome of Malvaceae plants. These differences may be related to the adaptive evolution process of different species [46]. This study found that the microvariation in the IR region of kenaf (*H. cannabinus*) chloroplast is slightly shorter than that of *Hibiscus syriacus*, which is not a random sequence difference, but may be due to the local contraction of the 12 bp segment of the *rps19* gene at the IRb LSC boundary—this change did not destroy the gene function, but instead reflected the conservative evolutionary strategy of Hibiscus plants to fine-tune the balance of genetic stability and adaptability through the IR region, and also explained the biological rationality that the differences in the IR region length of closely related species were mostly concentrated in the 10~15 bp range.

In addition, the maximum likelihood phylogenetic tree constructed based on shared CDS shows that kenaf (*H. cannabinus*) clusters with *Hibiscus sabdariffa* and *Urena procumbens* into one branch with a bootstrap value of 100%, which is consistent with the conclusion proposed by Tang et al. [19] that kenaf (*H. cannabinus*) has a close genetic relationship with *Hibiscus syriacus* and Abelmoschus esculentus; by increasing the coverage of species such as *Durio zibethinus* and *Theobroma cacao*, this study further clarified the phylogenetic position of kenaf (*H. cannabinus*) in the tribe *Hibisceae*.

The high-quality chloroplast (cp) genome sequence of kenaf (*H. cannabinus*) produced in this study and the related comparative evolutionary analysis provide key new insights for systematic and evolutionary studies of Malvaceae. Acting on core cp genes such as *rps2* and *rpoC2*, the identification of strong purification selection (Ka/Ks = 0.145 − 0.415) strengthened the conservative evolutionary pattern of “functional constraints on photosynthesis and transcription genes” in the Malvaceae family. The dynamic analysis of IR region boundaries, such as the cross-regional distribution of *rps19* at the LSC IRb junction, elucidates the fine structural variation mechanism of the *Hibisceae* tribe, jointly advancing the understanding of the evolutionary rules of CP genomes in terrestrial plants.

This study focused solely on analyzing the chloroplast (cp)-genome of one kenaf (*H. cannabinus*) genotype, without covering varieties of different geographical origins or uses—hindering full representation of the species’ cp-genome diversity, consistent with insufficient genotype coverage in prior studies. In evolutionary studies, coverage of close *Hibisceae* relatives remained incomplete, potentially compromising phylogenetic accuracy; future work can refine the kenaf (*H. cannabinus*) cp-genome research system by sequencing more genotypes, integrating multi-omics data, and expanding species coverage to support kenaf (*H. cannabinus*) genetic improvement and industrial development.

## Figures and Tables

**Table 1:** Statistics of chloroplast gene annotation information.

ID	Gene	tRNA	rRNA	mRNA	Pseudo
*Hibiscus_cannabinus*_L.	131	37	8	85	1

**Table 2:** Chloroplast gene functional classification.

Category	Gene Group	Gene Name
Photosynthesis	Subunits of photosystem I	*psaA*, *psaB*, *psaC*, *psaI*, *psaJ*
	Subunits of photosystem II	*psbA*, *psbB*, *psbC*, *psbD*, *psbE*, *psbF*, *psbH*, *psbI*, *psbJ*, *psbK*, *psbL*, *psbM*, *psbN*, *psbT*, *psbZ*
	Subunits of NADH dehydrogenase	*ndhA* *, *ndhB* * (*2*), *ndhC*, *ndhD*, *ndhE*, *ndhF*, *ndhG*, *ndhH*, *ndhI*, *ndhJ*, *ndhK*
	Subunits of cytochrome b/f complex	*petA*, *petB* *, *petD*, *petG*, *petL*, *petN*
	Subunits of ATP synthase	*atpA*, *atpB*, *atpE*, *atpF* *, *atpH*, *atpI*
	Large subunit of rubisco	*rbcL*
	Subunits of photochlorophyllide reductase	*-*
Self-replication	Proteins of large ribosomal subunit	*rpl14*, *rpl16* *, *rpl2* * (*2*), *rpl20*, *rpl22*, *rpl23*(*2*), *rpl32*, *rpl33*, *rpl36*
	Proteins of small ribosomal subunit	*rps11*, *rps12* ** (*2*), *rps14*, *rps15*, *rps16* *, *rps18*, *rps19*, *rps2*, *rps3*, *rps4*, *rps7*(*2*), *rps8*
	Subunits of RNA polymerase	*rpoA*, *rpoB*, *rpoC1* *, *rpoC2*
	Ribosomal RNAs	*rrn16*(*2*), *rrn23*(*2*), *rrn4.5*(*2*), *rrn5*(*2*)
	Transfer RNAs	*trnA-UGC* **(*2*), *trnC-GCA*, *trnD-GUC*, *trnE-UUC*, *trnF-GAA*, *trnG-GCC* *, *trnG-UCC*, *trnH-GUG*, *trnI-CAU*(*2*), *trnI-GAU* **(*2*), *trnK-UUU* *, *trnL-CAA*(*2*), *trnL-UAA* *, *trnL-UAG*, *trnM-CAU*, *trnN-GUU*(*2*), *trnP-UGG*, *trnQ-UUG*, *trnR-ACG*(*2*), *trnR-UCU*, *trnS-GCU*, *trnS-GGA*, *trnS-UGA*, *trnT-GGU*, *trnT-UGU*, *trnV-GAC*(*2*), *trnV-UAC* *, *trnW-CCA*, *trnY-GUA*, *trnfM-CAU*
Other genes	Maturase	*matK*
	Protease	*clpP* **
	Envelope membrane protein	*cemA*
	Acetyl-CoA carboxylase	*accD*
	C-type cytochrome synthesis gene	*ccsA*
	Translation initiation factor	*infA*
	other	*-*
Genes of unknown function	Conserved hypothetical chloroplast ORF	*#ycf1*, *ycf1*, *ycf2*(*2*), *ycf3* **, *ycf4*

Note: gene * indicates genes containing one intron; gene ** denotes genes with two introns; # Gene: Pseudogene; and gene (2) signifies genes with copy numbers > 1 (the specific copy number is shown in parentheses).

**Table 3:** Sequencing data statistics table.

Sample ID	ReadSum	BaseSum	GC (%)	Q20 (%)	Q30 (%)
*Hibiscus*_*cannabinus*_L.	19,641,502	5,892,450,600	37.31	98.30	92.40

**Table 4:** Chloroplast genome features.

Region	A Content/%	C Content/%	G Content/%	T Content/%	GC Content/%	Base Length/bp
LSC	32.04%	17.69%	16.66%	33.60%	34.35%	90,467
SSC	34.08%	16.14%	14.73%	35.05%	30.87%	19,486
IRa	28.74%	22.14%	20.47%	28.65%	42.61%	26,533
IRb	28.65%	20.47%	22.14%	28.74%	42.61%	26,533
Total	31.20%	18.68%	17.94%	32.18%	36.62%	163,019

**Table 5:** Analysis of RSCU in chloroplast genomes.

Amino Acids	Codon	Quantity	RSCU	Amino Acids	Codon	Quantity	RSCU	Amino Acids	Codon	Quantity	RSCU
Ter	UAA	43	1.6329	Lys	AAA	893	1.5136	Arg	AGG	147	0.642
Ter	UAG	19	0.7215	Lys	AAG	287	0.4864	Arg	CGA	314	1.371
Ter	UGA	17	0.6456	Leu	CUA	320	0.7968	Arg	CGC	110	0.4806
Ala	GCA	338	1.0416	Leu	CUC	154	0.3834	Arg	CGG	95	0.4146
Ala	GCC	214	0.6596	Leu	CUG	148	0.3684	Arg	CGU	297	1.2972
Ala	GCG	169	0.5208	Leu	CUU	494	1.23	Ser	AGC	97	0.3426
Ala	GCU	577	1.778	Leu	UUA	801	1.9944	Ser	AGU	353	1.2456
Cys	UGC	59	0.4646	Leu	UUG	493	1.2276	Ser	UCA	362	1.2774
Cys	UGU	195	1.5354	Met	AUA	0	0	Ser	UCC	256	0.9036
Asp	GAC	185	0.4062	Met	AUC	0	0	Ser	UCG	141	0.4974
Asp	GAU	726	1.5938	Met	AUG	528	7	Ser	UCU	491	1.7328
Glu	GAA	899	1.5072	Met	AUU	0	0	Thr	ACA	352	1.2064
Glu	GAG	294	0.4928	Met	CUG	0	0	Thr	ACC	227	0.778
Phe	UUC	423	0.6594	Met	GUG	0	0	Thr	ACG	137	0.4696
Phe	UUU	860	1.3406	Met	UUG	0	0	Thr	ACU	451	1.546
Gly	GGA	620	1.5404	Asn	AAC	255	0.4674	Val	GUA	473	1.5112
Gly	GGC	171	0.4248	Asn	AAU	836	1.5326	Val	GUC	151	0.4824
Gly	GGG	290	0.7204	Pro	CCA	264	1.1272	Val	GUG	169	0.54
Gly	GGU	529	1.3144	Pro	CCC	177	0.7556	Val	GUU	459	1.4664
His	CAC	141	0.509	Pro	CCG	131	0.5592	Trp	UGG	398	1
His	CAU	413	1.491	Pro	CCU	365	1.558	Tyr	UAC	168	0.3906
Ile	AUA	626	0.9498	Gln	CAA	637	1.5536	Tyr	UAU	692	1.6094
Ile	AUC	371	0.5631	Gln	CAG	183	0.4464				
Ile	AUU	980	1.4871	Arg	AGA	411	1.7946				

**Table 6:** Statistical table of the Ka/Ks analysis of chloroplast genes.

Gene	Ka/Ks	*p*-Value
*Hibiscus*_*cannabinus*_L._rps2 vs. MK792856_rps2	0.145281	0.00117837
*Hibiscus*_*cannabinus*_L._rpoC2 vs. MK792856_rpoC2	0.414721	5.56441 × 10^−6^
*Hibiscus*_*cannabinus*_L._rpoC1 vs. MK792856_rpoC1	0.248324	5.35013 × 10^−5^
*Hibiscus*_*cannabinus*_L._rpoB vs. MK792856_rpoB	0.195518	1.90321 × 10^−9^

## Data Availability

The original contributions presented in this study are included in the article. Further inquiries can be directed to the corresponding authors. All original data (including sequencing reads and annotated genomes) supporting the reported results have been submitted to NCBI GenBank PX646974.

## Appendix A

**Table A1:** Reads re-sequencing genomic results statistics table.

ID	Mapping_Reads_Number	Average_Coverage	Insert_Size
*Hibiscus_cannabinus*_L.	1,932,666	3701.037	323.81 ± 75.26
